# Depressive symptoms and use of health services among older adults in Israel

**DOI:** 10.1186/s13584-020-00374-5

**Published:** 2020-06-02

**Authors:** Netta Bentur, Anthony David Heymann

**Affiliations:** 1 Stanley Steyer School for Health Professionals, Tel-Aviv University, Tel Aviv, Israel; 2 The Department of Family Medicine, The Sackler School of Medicine, University of Tel Aviv, Tel Aviv, Israel

**Keywords:** Depressive symptoms, Older adults, Point prevalence, Israel

## Abstract

**Objectives:**

Depressive symptoms are often undetected, particularly among older adults. The purpose of this study is to provide information on the prevalence, characteristics, and patterns of depressive symptoms among older adults residing in the community in Israel, and their health-care utilization.

**Methods:**

A cross-sectional survey was conducted among a random sample of 2502 members of one HMO in Israel, aged 65+. They were interviewed by telephone with the GDS-15 scale, which serves as the gold standard for depressive symptoms. Data from the computerized medical records of the HMO were added to the interview file, including the diagnosis of depression, purchase of antidepressant medication and use of services.

**Results:**

The average age of respondents was 73; 54% were women. They tended to be older, living alone, suffering from falls and from sleep disorders, and to have poor subjective health status. 24% scored 6+ on the GDS scale. A significant association was found between a GDS score of 6+ and increased hospitalizations, visits to the emergency room and/or to family physicians and specialists.

**Conclusion:**

We found a high prevalence of depression. Its negative effects on the individual and increased costs to the health system, supports the screening and treatment of the disease in the older population. This problem should be a national priority, with screening and treatment becoming part of the national quality of care indicators which would then be implemented by the HMOs as part of an integrated disease management program for the elderly.

## Background

Depressive symptoms often lead to disability and yet are often undetected and under treated among older adults [1–3]. It has been found to be associated with increased physical symptoms, functional decline [4] and decreased quality of life [5, 6]. It may also be detrimental to relations with family and the immediate milieu, and the cause of much suffering [7].

Late-life depression, according to DSM-V and ICD-10, refers to depressive syndromes that appear in adults aged 65+. Most depression among older adults presents as mild depression and minor depressive conditions such as dysthymic disorders, such as an altered sense of self and interpersonal interactions or depressed mood [8]. These symptoms are reportedly more common among older adults than is the full syndrome of major depression but they are nevertheless associated with significant morbidity and can cause significant impairment [1, 9, 10].

Studies have reported a prevalence of late-life depression and depressive disorders which vary considerably and range from 4.5 to 37% [2, 11–15]. This derives from differences in the disease definition, the time-frame, differences in the composition of the population studied (entire population, limited by age group, ethnicity) and methods of study and analysis.

Studies have also identified risk factors for symptoms of late-life depression that include demographic characteristics, such as female gender and higher age [13, 15–19], chronic medical illnesses [20], physical impairment, frailty, poor self-rated health [21, 22], and distressing family and life events [21, 23, 24].

Prolonged depressive symptoms result in high costs to the health system and are associated with substantial costs arising from diagnostic procedures and from the treatment of depression. This amounts to a substantial national economic burden [25–28]. Bock et al. [29] found that in Germany the costs for patients with depression exceeded costs of those without by a factor of 2.6. In the US, Medicare recipients with a history of depression had significantly higher medical costs than recipients with no history of depression [30]. Some of these costs might have been avoided had there been appropriate diagnosis and treatment.

Although studies around the world have yielded data on the prevalence and characteristics of depressive symptoms among older adults, findings cannot necessarily be generalized from one country to all countries. In Israel, the only information on prevalence of depression among older adults was published by Biderman et al. in 2003 [31], reporting that 17% of community-resident older adults had been diagnosed with depression in the course of the preceding year. The purpose of the present study was to provide updated information on the prevalence and characteristics of depression among community-dwelling older adults in Israel, and their patterns of health-care use.

## The study method

Study design: A cross-sectional survey was conducted in Maccabi Healthcare Services, the second largest Health Maintenance Organization (HMO) in Israel.

Study Population: The target population numbered 4350 people aged 65+ with no recorded diagnosis of dementia, who were randomly sampled from HMO patient records. Of these, 2502 (58%) were surveyed. The main reasons for non-participation were refusal to be interviewed (19%), lack of ability to cooperate with a telephone interview because of hearing or language problems and discontinued the interview (23%). 79% were interviewed in Hebrew, 17% in Russian and 4% in English or Spanish.

The Study Procedures: Telephone interviews were conducted using a computerized questionnaire. All the interviewers were experienced in such surveys and were trained to undertake this study after being fully informed of its purpose. We supervised the interviewers closely. The quality of the interviews was assessed and immediate feedback given to the interviewers.

### Study instruments

Depressive symptoms were assessed using a Hebrew version of the 15-item Geriatric Depression Scale GDS-15 [32], which had been previously validated for telephone interviews in Hebrew [33]. To stratify the analyses into two groups, a score of 6 or more was used as the cut-off point for significant depressive symptoms. This score has been found to yield a cut-off of high sensitivity and specificity in a study of older Israelis living in the community [34] as well as in other studies [35, 36]. We also evaluated the reliability of the Hebrew GDS as a telephone tool by means of face-to-face and telephone interviews of older adults aged 65+, one or 2 weeks apart. The internal reliability using Cronbach’s alpha coefficient was 0.88 for the telephone interviews preceding face-to-face interview and 0.89 for interviews in the reverse order (not published).

The computerized telephone questionnaire also included questions on the following topics: subjective health status, sleep disorders, and demographic information. Administrative data from the computerized medical records of the HMO were added to the interview file. These data included information on diagnosis of depression and purchase of antidepressant medication in the previous year, details of the previous year’s hospitalizations, visits to the emergency room and to family physicians. The study was approved by the IRB committee of Maccabi Healthcare Services.

Statistical analysis: The 12-month prevalence of depression symptoms was described using means, Standard Deviation and Confidence Intervals. Bivariate analysis was used to compare the characteristics of the population according to the GDS cut-off point, and multivariate logistic regression models were used to identify the characteristics of older adults that were independently related to depressive symptoms, hospitalization, and emergency-room visits.

## Results

About 54% of the study population were women; the average age was 73 (SD = 6), 36% were aged 75+. About a quarter (24%) scored GDS ≥ 6 on the GDS scale (598 people); of these, 19% scored 6–10 and 5% scored 11–15. The characteristics of the study population according to GDS score are presented in Table 1.

**Table 1:** Characteristics of the Study Population, by GDS Score

	Total Population	GDS ≥ 6	GDS < 6
N	(*N* = 2502)	(*N* = 598)	(*N* = 1904)
%	100%	24%	76%
Women*	54	67	51
**Age** *			
65–69	34	26	37
70–74	30	28	31
75–80	23	27	21
81+	13	18	11
Lives alone or with caregiver*	26	37	22
Education level*			
up to 8 years of schooling	14 26	22	12
up to 12 years of schooling	3331	31	34
13+ years of schooling	52 43	47	55
Fell in the past half-year*	25	37	22
Health condition today worse/much worse than last year*	43	65	38
Sleep disorder/s*	46 55	67	41
**Hospitalization in past year**	18	11	19
**Visits to emergency room in past year**	17 22	11	18

* *p* < 0.01

A diagnosis of depression in the previous year was found in 7% of the medical records of the total population. 49% of these depressed patients had a GDS score of 6 or more in the telephone interview, and 51% had a lower score. A diagnosis of depression or a record of purchase of antidepressant in the previous year, were found in the medical records of only 15% of patients.

Using logistic regression where the dependent variable was GDS (GDS ≥ 6/ GDS < 6) and the characteristics of older adults were the independent variables, a significant independent association was found between a GDS ≥ 6 and the following variables: Being female, older, living alone, having had a fall in the past 6 months, sleep disorder, and health status perceived as deteriorating by the patient (Table 2).

**Table 2:** Logistic Regression Analysis, to explain the Characteristics of Older adults with Depressive Symptoms (GDS ≥ 6)

	Odds Ratio Exp(B)	95% C.I. Lower Upper	
Women (vs. men)	1.287	1.021	1.622
Older	0.964	0.945	0.983
Living alone (vs. with others)	1.436	1.126	1.830
Fell in the past half year	1.374	1.075	1.757
Disturbed sleep	2.565	2.056	3.201
Less favorable assessment of health than in the past (vs. same or better)	2.251	1.797	2.820

*p < 0.05

More older adults with GDS ≥ 6 than those with GDS < 6 were hospitalized at least once in the previous year (19 and 11% respectively, *p* < 0.05), visited the emergency room (ER) without being hospitalized (18 and 11% respectively, *p* < 0.05), and visited their primary physicians (15 and 12 times respectively, *p* < 0.05). In a logistic regression analysis with hospitalization in the previous year as the dependent variable and the characteristics of older adults were the independent variable, a significant independent association was found with the following variables: GDS ≥ 6, female sex, living alone, and health status self-perceived as deteriorating (Table 3).

**Table 3:** Logistic Regression Analysis, to explain Hospitalization and ER visits of older adults

	At Least 1 Hospitalization during the Year			At Least 1 Visit to ER in the Past Year		
	Odds Ratio	95% C.I		Odds Ratio	95% C.I	
		Lower	Upper		Lower	Upper
GDS6+	1.657	0.505	2.001	1.446	1.095	1.908
Women	0.619	−0.479	0.825			
Living alone	1.616	1.291	2.244	1.390	1.066	1.810
Less favorable assessment of health than in the past (vs. same or better)	1.597	1.137	2.103	1.553	1.202	2.008

* *p* < 0.01

In a logistic regression analysis in which ER visit in the previous year was the dependent variable and the characteristics of older adults were the independent variable, a significant independent association was found with the following variables: GDS ≥ 6, living alone, and a health status perceived as deteriorating (Table 3). More visits to family physicians were found in a multiple regression analysis to be independently associated with GDS ≥ 6, living alone, and health status perceived as deteriorating (not in table).

## Discussion

The study provides information on the 12-month prevalence of depressive symptoms among a sample of community-dwelling people aged 65+ in Israel, and identifies their characteristics and use of healthcare services. About a quarter of the study population suffered depressive symptoms. Our results seem to fall in the middle range of prevalence studies found in other countries [2, 11]. In a meta-analysis of studies on the prevalence of depressive symptoms in Chinese older adults the overall prevalence of depressive was found to be 23.6% [15]. In another meta-analysis the prevalence of depressive disorders among older adults ranged from 4.5 to 37.4%; the pooled prevalence was 17.1% [12]. The researchers concluded that potential sources of high heterogeneity of prevalence were study design, sampling strategy, study quality, and diagnostic criteria.

Our findings regarding the characteristics of older adults suffering from depressive symptoms reinforce those of other studies regarding the risk factors of being female, being older, and living alone [13–22]. We showed that depressive symptoms are associated with an increased disability burden in older adults. This finding of a positive association between depression and frailty in cross-sectional studies has been reported previously [22, 37]. This study also supports previous research that found an independent association between perceived deteriorating health and depression [38] and between falls and depression [39].

Among elderly people with depressive symptoms we found increased outpatient resource utilization and more visits to family physicians as well as hospitalization which have also been previously reported [28, 40]. Depression may increase medical symptoms and generate a greater disease burden, which may lead to more use of medical services. Possibly, older adults may perceive depression as a normal reaction to their physical problems, social isolation, and other difficulties, and may have stigmatized attitudes towards mental problems, discouraging them from raising mental health issues with their physicians.

Limitations: This study has several limitations. There is a selection bias due to the fact that the interviews were conducted only among Jewish members of the HMO. Another potential bias might be the fact that we do not know the reasons for refusal to be interviewed. In addition, the fact that hearing and language problems constituted exclusion criteria might generate bias, as hearing problems may be related to higher rates of depression symptoms among older adults [41]. In addition, we did not assess co-morbidity, but only excluded people with diagnoses of dementia.

With a progressively ageing population identification and treatment of depression in older adults becomes increasingly important. Steps should therefore be taken to both improve case-finding among primary physicians and nurses and to raise the awareness of older adults about depressive symptoms and the availability of treatment. Israel has a very efficient primary care healthcare system which has the potential for improving the identification of elderly patients with depression. The first step should be to make this a national priority and make depression screening in this age group a national quality indicator. This would encourage the four Israeli HMOs to approach this disease like any other and use standard disease management tools. This requires the creation of a computerized disease register, provision of appropriate diagnostic instruments, training of medical staff, feedback to physicians and nurses, and raising awareness among the relevant patient population and their caregivers. An example of a successful national model of psychiatric screening is that of post-natal depression identification. A universal screening program was mandated in 2013 (Israel Ministry of Health, Directive No. 20/12, 2012), and has since been implemented in all Mother-and-Child Health Clinics by public health nurses. In a similar manner the HMOs could organize screening for depression in all elderly members, or focus the screening on specific segments, such as patients after hospital discharge or those with frailty or significant comorbidity. From a technical point of view identification of these groups should be relatively easy, using the extensive databases of each HMO. Such a decision would entail committing resources and ensuring that all identified patients are offered treatment. As with the post-partum screening, the success of such a program would rely on proper training of the primary care team [42]. The GDS can be administered by telephone, so a HMO call center could be set up to undertake this task. An alternative approach might be sending electronic messages to elderly HMO members encouraging them to enter the web site or phone applications of the HMO in order to do a yearly screening test. Another approach might be to widen the scope of screening within this population and include the assessment of depressive symptoms within a broader geriatric assessment which should include physical, nutritional, social and medication assessment. One needs to keep in mind that the GDS is a screening tool for depressive symptoms; hence there is also a need for clinical confirmation of a diagnosis of depression.

## Conclusion

One of every four older adults in Israel suffers from significant depressive symptoms. This is likely to negatively affect their lives and those of their families, and give rise to substantial costs to the health care system. This must be a national priority and become part of the quality of care indicators in the community. The approach can be focused on depression alone or as part of a broader geriatric assessment. Which could then be implemented by the Israeli HMOs as part of an integrated disease management program for the elderly.

## Data Availability

The dataset is available from author upon request.
